# Bilateral Obturator Hernia Diagnosed by Computed Tomography: A Case Report with Review of the Literature

**DOI:** 10.1155/2014/625873

**Published:** 2014-12-03

**Authors:** Sanjay M. Khaladkar, Anubhav Kamal, Sahil Garg, Vigyat Kamal

**Affiliations:** Department of Radio-Diagnosis, Dr. D.Y. Patil Medical College, Pimpri, Pune 411018, India

## Abstract

Obturator hernia is a rare form of abdominal hernia and a diagnostic challenge. It is commonly seen in elderly thin females. Its diagnosis is often delayed with resultant increased morbidity and mortality due to bowel ischemia/gangrene. It is mistakenly diagnosed as femoral or inguinal hernia on USG. Computed tomography is diagnostic and is a valuable tool for preoperative diagnosis. This report presents a case of 70-year-old thin female presenting with intestinal obstruction due to left sided obstructed obturator hernia. USG showed small bowel obstruction and an obstructed left sided femoral hernia. CT scan of abdomen and pelvis with inguinal and upper thigh region disclosed left sided obturator hernia. It also detected clinically occult right sided obturator hernia. Early diagnosis and surgical treatment contribute greatly in reducing the morbidity and mortality rate.

## 1. Introduction

An obturator hernia is a rare cause of all abdominal wall hernias commonly seen in females. Its clinical diagnosis is often difficult due to uncommon incidence, its deep location, and infrequent symptoms and signs. Delay in its diagnosis causes poor prognosis with increased morbidity. Early CT imaging establishes diagnosis and detects asymptomatic contralateral obturator hernia. The following case report highlights these diagnostic difficulties and reviews the current literature on diagnosis and management of such cases.

## 2. Case Report

**Figure 1:**
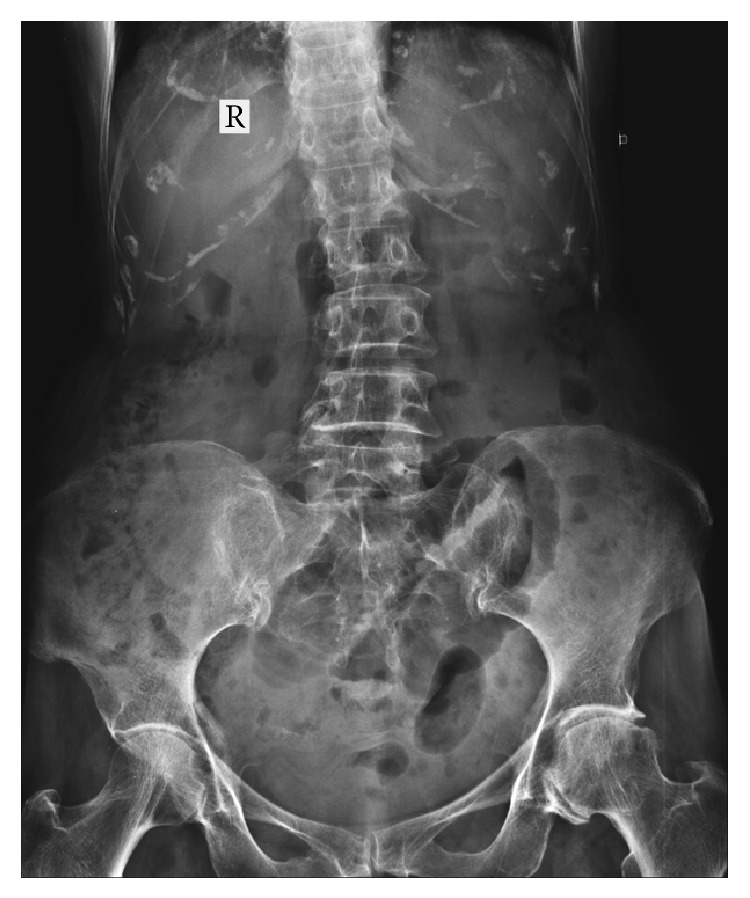
X-ray standing abdomen showing dilated small bowel loops in lower abdomen and pelvis.

**Figure 2:**
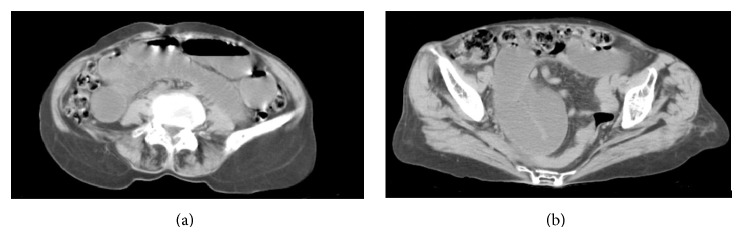
Plain CT scan of lower abdomen and pelvis showing dilated small bowel loops in abdomen and pelvis.

**Figure 3:**
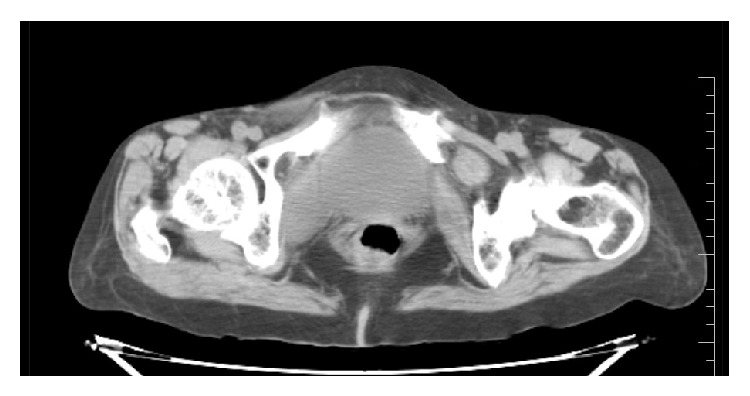
CT scan of lower pelvis and inguinal region showing herniation of small bowel loop through left obturator canal extending between pectineus and obturator internus muscles.

**Figure 4:**
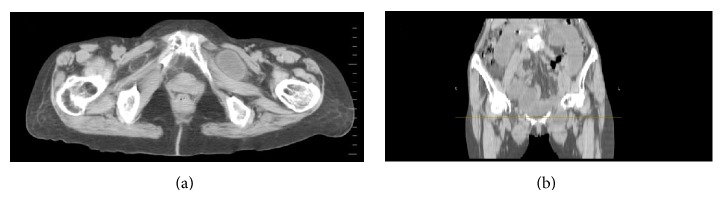
(a) Plain CT scan of inguinal and upper thigh region showing herniated small bowel loop in left obturator canal between pectineus and obturator externus muscles and herniation of mesentery in right obturator canal. (b) Coronal reformatted image at level of obturator canal showing herniated small bowel loop in left obturator canal and herniation of mesentery in right obturator canal.

70-year-old known hypertensive female patient presented with intermittent abdominal pain and vomiting for 2 days. She gave past history of pulmonary Koch's 10 years back for which she completed AKT. On examination the patient was thin-built, conscious, and well oriented. Blood pressure was 150/90 mmHg. Respiratory rate was 22/min. Per abdomen examination showed mild abdominal distension. She was referred for USG Abdomen and Pelvis. USG Abdomen and Pelvis showed mild dilatation of small bowel loops in entire abdomen (caliber = 3–3.5 cm) with intermittent to-and-fro peristalsis. Mild free fluid was noted in pelvis and in between small bowel loops. Left inguinal and left upper thigh region showed a herniated small bowel loop extending in medial aspect of upper thigh which was irreducible. A diagnosis of obstructed and irreducible left femoral hernia was made. X-ray standing abdomen (Figure 1) revealed dilated small bowel loops in mid and lower abdomen with no pneumoperitoneum. She was referred for emergency plain CT scan of abdomen and pelvis. Small bowel loops in abdomen and pelvis appeared fluid-filled and dilated of caliber 3–3.5 cms (Figures 2(a) and 2(b)). There was herniation of a bowel loop of length 3.5 cm through left obturator foramen extending inferiorly between pectineus muscle anteriorly and obturator externus muscle posteriorly, suggestive of obturator hernia (Figures 3, 4(a), and 4(b)). Left pectineus muscle was compressed and displaced anteriorly. Few small bowel loops appeared collapsed; hence obstruction was likely to be at distal jejunum/proximal ileal level. Visualized colon appeared collapsed. Also, hernia of omentum/mesentery was noted from the right obturator foramen measuring approximately 2 × 0.9 cm. Herniated omentum/mesentery was seen to lie in between the pectineus muscle anteriorly and obturator externus muscle posteriorly (Figures 4(a) and 4(b)). No herniated bowel loop was seen through right obturator foramen in the present study. A diagnosis of obstructed left obturator hernia with proximal dilatation of small bowel loops and right obturator hernia containing omentum/mesentery was made.

Exploratory laparotomy was performed which confirmed obstructed left obturator hernia and the entrapped segment of bowel was released. Small bowel 100 cm proximal to ileocaecal junction was found to be gangrenous. Hence, resection anastomosis was done. Approximately 60 cm of small bowel was resected. Obturator hernia was also confirmed on right side containing mesentery. The hernial defect was covered on either side with prosthetic mesh. Care was taken not to damage the obturator nerve on either side while covering it with prosthetic mesh.

## 3. Discussion

Obturator hernias account for 0.07–1% of all hernias and 0.2–1.6% of all cases of mechanical obstruction of small bowel (Table 1). They have the highest mortality rate of all abdominal wall hernias (13–40%) with female predominance and with female to male ratio of 6 : 1. Female preponderance is due to large and more oblique incline of obturator canal in female pelvis. It occurs more frequently on right side as sigmoid colon overlies left obturator foramen. Bilateral obturator hernias are seen in 6% of cases [1].

It is commonly seen in elderly females and postpregnancy patients due to greater width of the pelvis, larger obturator canal, and increased laxity of the pelvic tissues. It is nicknamed “little old lady's hernia” as it affects this group due to atrophy and loss of preperitoneal fat around obturator vessels in obturator canal predisposing to obturator hernia. Multiparity, COPD, constipation, ascites, and causes of raised intra-abdominal pressure are its other predisposing factors [2].

Arnaud de Ronsil was the first to describe the obturator hernia in 1724 and Obre was the first to perform the successful operation in 1851 [1].

Obturator hernia occurs through obturator canal which is 1 cm wide and 2-3 cm long. The obturator foramen is formed by continuity of pubic and ischial bones and is covered by obturator membrane except in its anterosuperior aspect where it is perforated by obturator nerve, artery, and vein. These travel along 2-3 cm in oblique tunnel (obturator canal) formed by obturator externus and internus muscles. Peritoneal hernia develops through this defect by first increasing separation of muscular band of obturator internus muscle and later separating obturator externus muscle. The hernia sac finally lies on top of obturator externus muscle beneath pectineus muscle.

Clinical diagnosis of obturator hernia is difficult due to uncommon incidence, deep location, and infrequent symptoms and signs. Early diagnosis is needed as delay in its recognition causes poor prognosis in patients. Vast majority of patients fail to complain of obturator neuralgia. Physical examination is not contributory with resultant delay in diagnosis. Hernial sac irritates and compresses obturator nerve within the canal causing medial thigh pain (Howship-Romberg sign). Howship-Romberg sign is pain radiating down the medial aspect of the thigh to the knee and less often to the hip due to compression of the anterior division of the obturator nerve. It is considered pathognomonic of obturator hernia and is seen in up to 15–50% of patients. Palpable mass is found in 20% of cases in the proximal medial aspect of the thigh at the origin of the adductor muscles [1, 3]. High index of suspicion of obturator hernia should be made when an elderly patient presents with small bowel obstruction with intermittent symptoms and medial thigh pain is present. Early and rapid clinical and appropriate radiological evaluation followed by early surgery is essential for successful treatment. Delay in specific diagnosis causes increased morbidity and mortality as the only treatment available is surgical reduction and repair of hernia. Surgical intervention is delayed due to clinical and radiological diagnostic difficulty.

Early CT imaging causes early diagnosis with reduced morbidity and mortality associated with obturator hernia [4]. X-ray standing abdomen shows evidence of small bowel obstruction in cases of obstructed obturator hernia. USG shows hernia in inguinal and upper femoral region. Often it is misdiagnosed as inguinal or femoral hernia. Signs of small bowel obstruction are seen if it is obstructed and incarcerated. CT scan is diagnostic (Table 2). CT imaging of bowel herniating through the obturator foramen and lying between the pectineus muscle anteriorly and obturator externus muscle posteriorly is diagnostic. This is best demonstrated by low axial CT image in inguinal and upper thigh region. CT can also diagnose asymptomatic bilateral obturator hernia [5].

Various surgical approaches are described in the literature in cases of obstructed obturator hernia. Abdominal, inguinal, retropubic, obturator, and laparoscopic approaches have been described. The published data favors the abdominal approach, utilizing a low midline incision. This method allows the surgeon to establish the diagnosis, avoid obturator vessels, give better exposure of the obturator ring, and facilitate bowel resection if necessary. Herniorrhaphy is performed by simple closure of the hernial defect with interrupted sutures, placement of a synthetic mesh. These have the lowest complication rates. Laparoscopic repair of obturator hernia can also be done. It produces less postoperative pain with shorter hospital stay and fewer pulmonary complications [6, 7].

## 4. Conclusion

High index of suspicion for obturator hernia should be made in a patient presenting with small bowel obstruction with medial thigh pain. All hernia orifices (inguinal, femoral) should be assessed and screening for Howship-Romberg sign should be done. Early diagnosis can be done by early CT scanning after ruling out inguinal and femoral hernias. This will increase the speed of diagnosis and avoid complications like bowel ischemia.

## Useful Tips and Tricks during Examination and Imaging of Obturator Hernia

*Clinical Features*

- Emaciated thin elderly females.
- Intermittent attacks of small bowel obstruction.
- Howship-Romberg sign.
- Referred pain relieved by flexion of thigh and aggravated by extension, abduction, and medial rotation of thigh.
- Tender swelling in region of obturator foramen on vaginal and rectal examination.

*X-Ray*

- Small bowel obstruction.
- Gas shadow in obturator foramen area.

*USG*

- Hernia deep to pectineus muscle on axial scan of inguinal region.
- Hernia occurring in plane similar to femoral canal but deep to pubic ramus.
- Small bowel obstruction.

*CT*

- Identification of hernia between pectineus and obturator externus muscles.
- Small bowel obstruction.

## Figures and Tables

**Table 1:** Hernia types with typical location and diagnostic imaging findings.

Hernia name	Location	Diagnostic imaging features
Inguinal direct	Hesselbach's triangle	Medial to the inferior epigastric artery (MD)

Inguinal indirect	Hesselbach's triangle	Lateral to the inferior epigastric artery (IL)

Pantaloon	Hesselbach's triangle	Contains both direct and indirect inguinal hernias

Spigelian	Along linea semilunaris	At junction of lateral abdominal muscles and rectus sheath

Paraumbilical	Defect in the linea alba	Associated with diastasis of the rectus muscles

Femoral	Medial aspect of the femoral canal	Hernia sac with femoral vein compression

De Garengeot	Femoral canal	Contains the appendix

Amyand	Inguinal canal	Contains the appendix

Littre	Any location	Contains Meckel's diverticulum

Richter	Any location, though usually along anterior abdominal wall	Contains only antimesenteric side of a loop of bowel

Obturator	Obturator canal through obturator foramen	Between pectineus and obturator externus muscles; often presents with incarceration

Grynfeltt-Lesshaft	Upper lumbar triangle	Location

Petit	Lower lumbar triangle	Location

**Table 2:** Advantages, limitations, and pitfalls of diagnostic modalities for abdominal wall hernias.

Modality	Advantages	Limitations	Pitfalls
USG	Availability, portability, low cost, and no ionizing radiation Capability of real time imaging (DASH, dynamic abdominal sonography for hernia, in supine and upright position at rest and with Valsalva's maneuver), comparison with unaffected side Can diagnose postoperative seromas and hematomas and can diagnose recurrent hernia along edge of repair using Valsalva maneuver	Being operator dependent, need of high frequency transducer, obesity, scarring, and patients with acute abdominal pain Sonographic evaluation after hernia repair may be difficult due to dense shadowing caused by surgical mesh	Lipoma of spermatic cord and abdominal wall

MDCT	High spatial and contrast resolution and multiplanar imaging Detection of defect and contents of hernia and its complications (like incarceration, strangulation, bowel ischemia, and bowel obstruction) Detection of postoperative complications—seroma, hematoma Differentiation of hernia from other abdominal wall masses like hematoma, abscess, tumor, and undescended testis CT performed during postural maneuver (prone, lateral decubitus position), maneuver to increase intra-abdominal pressure (straining, Valsalva's maneuver) and increase lesion detection	Ionizing radiation, pregnant patients, being expensive, and availability	
